# Welcomed Pregnancies: Characteristics and Patterns of Adjusting to Unwanted, Unplanned, Untimely or Otherwise Difficult Pregnancies

**DOI:** 10.7759/cureus.61885

**Published:** 2024-06-07

**Authors:** David C Reardon

**Affiliations:** 1 Research, Elliot Institute, St. Peters, USA; 2 Research, Charlotte Lozier Institute, Arlington, USA

**Keywords:** risk factors, abortion decision making, pregnancy help centers, unwanted pregnancies, unplanned pregnancies, pregnancy counseling

## Abstract

Background

Women facing problematic pregnancies, defined as “unplanned, mistimed, unwanted, or otherwise difficult,” either have abortions or make adjustments to welcome these pregnancies. These adjustments are understudied. Pregnancy resource centers that provide counseling and services to assist in the process of welcoming pregnancies have been the focus of controversy due to their refusal to counsel or refer for abortions. This survey of a national population of women seeks to quantify changes in attitudes toward problematic pregnancies that are not aborted and to gauge levels of contact with pregnancy help centers and perceptions of harm or benefits attributed to those contacts.

Methodology

A national research firm was enlisted to obtain 1,000 surveys completed by female residents of the United States aged 41-45, inclusive. Women reporting a history of abortion were surveyed along one path. For those who did not have abortions but reported a problematic pregnancy, questions were presented to assess changes in attitude toward their pregnancy from the date they first learned they were pregnant to 90 days later, their considerations of abortion, whether they had contact with a pregnancy help center, and their assessment of that contact on either harming or improving their lives.

Results

Among 275 respondents who had no history of abortion but had ultimately welcomed a problematic pregnancy, 112 (40.7%) had been at higher risk of abortion. Positive attitudes toward their pregnancies increased most rapidly for women who had been at higher risk of abortion but were lower on the day they first learned they were pregnant. Overall, 34 (12.4%) reported they had contacted a pregnancy help center that did not refer for abortions. Another 37 (13.5%) were uncertain if they had contacted an organization fitting that description. Both groups reported the contact improved their lives, on average. Negative assessments were uncommon and all were of a small degree.

Conclusions

Women facing problematic pregnancies who did not choose abortion experienced rapid improvements in feelings of wantedness, timeliness, acceptance, welcoming, and desirability toward the pregnancy. The rate of improvement was most rapid among those who had investigated and considered abortion. Women reporting contact with pregnancy help centers almost always assess it as having improved their lives.

## Introduction

Many, perhaps even most pregnancies, are unplanned, untimely, or unwanted [1,2]. Some are subject to induced abortions. Others are carried to term after consideration of abortion. It is also known that women’s feelings about these pregnancies may change rapidly and significantly over time [3-6]. In the face of strong evidence that many women attach multiple layers of meaning and value to such pregnancies [7], there is rising concern about the validity of overgeneralizing terms such as “unwanted” or “unintended” [7-9].

Recognizing this fact, a large number of pregnancy help centers exist to help women “choose life” rather than abortion [10]. According to a four-week follow-up survey of 857 pregnant women considering abortion who were identified through Google Ads, 260 (30.3%) reported that they may have had contact with a pregnancy help center, of whom 112 (13.1%) could be confirmed for having contact with a pregnancy resource center that did not refer for abortions [11]. Those who reported contact with such centers were approximately half as likely to continue seeking an abortion [11]. Another study, in contrast, reported that only seven out of 114 abortion patients (6.1%) had contacted a pregnancy help center, of whom only three had been considering an abortion at the time of their visit [12]. While the work of these organizations has been criticized by abortion rights advocates as deceptive and harmful to women [13-15], we can find no studies drawn from the general population examining women’s own evaluations of their experiences with these organizations.

In this retrospective descriptive study, we sought to characterize changes in women’s feelings regarding problematic pregnancies, defined herein as any pregnancy self-described as “unplanned, mistimed, unwanted, or otherwise difficult.” The subset of those pregnancies that are successfully carried to term is hereinafter referred to as welcomed pregnancies.

Our hypothesis is that women’s feelings regarding a problematic pregnancy will tend to grow more positive during the course of the pregnancy, at least for women whose pregnancies become welcomed pregnancies. We therefore sought to measure women’s retrospective evaluation of changes in their feelings toward their welcomed pregnancies between the time they first learned they were pregnant and three months later. Within this group of women, we also sought to identify differences associated with women who considered abortion and those who did not. Finally, we also sought to examine the rate of contact with pregnancy help centers among women with welcomed pregnancies and to gauge the degree of helpfulness or harm that women attributed to that experience.

## Materials and methods

A total of 1,000 completed interviews with females aged 41 to 45, inclusive, were collected using the Cint.com market research services at a cost of three dollars ($3.00) per completed survey. The respondents were randomly drawn from a subset of the 28 million U.S. residents in the Cint.com survey panel who were identified as females 41-45 years of age, inclusive. Most panel members have previously completed surveys for Cint clients. In our case, the invitation to prospective respondents did not identify the survey topic but did specify that respondents would receive a small incentive determined by Cint, such as credit toward a gift card or online access to something of value upon completion of the survey, drawn from the amount paid to Cint for its survey distribution services.

Following a short series of demographic questions, women were asked about their reproductive history. Women who reported an induced abortion were taken down a questionnaire path investigating their abortion experiences, reported elsewhere [16,17]. Women who reported no history of abortion but did report a history of at least one “unplanned, mistimed, unwanted, or otherwise difficult pregnancy” were presented with the questions reported herein. Notably, the inclusion of “otherwise difficult” pregnancies intentionally captured planned pregnancies, which may have become problematic due to subsequent medical, social, or economic conditions that may have impacted abortion risk and/or interest in pregnancy center resources.

Within the survey branching, the paths of women reporting abortion and those reporting problematic pregnancies were mutually exclusive. More specifically, women who reported a history of abortion were not offered the questions found in the welcomed pregnancies path in order to keep the number of questions required to complete the survey similar for both paths.

Respondents on the welcomed pregnancies path were asked to rate their reactions both on the day they first learned they were pregnant and 90 days later. These assessments were made on visual analog scales labeled from Untimely to Timely, Unwanted to Wanted, Rejected to Accepted, Undesirable to Desirable, and Very Unwelcome to Very Welcome. Each scale was presented as a slider on a visual analog scale without any hash marks, only the abovenamed labels at either end of the scale. For the purposes of parametric analyses, the selected position of the slider was electronically coded on a 101-point scale from 0 to 100, inclusive.

Abortion risk was assessed using three similarly provisioned visual analog scales. Respondents rated their agreement from Not at All to Very Much So to three statements: “I considered having an abortion during one of my pregnancies”; “I investigated either where to get an abortion or the cost”; and “If I had not had support from others, I may have had an abortion.” Women were classified as being at higher risk of abortion if their answer was over 50 on any one or more of the three 0-100 point abortion risk scales.

The outcome of the pregnancy was then recorded with five dichotomous options of “healthy live birth,” “birth with newborn defects,” “miscarriage, stillbirth or other natural loss,” “abortion,” and “other.” Respondents were then presented with another visual analog scale asking them to rank their response to the statement “Today, the outcome of that pregnancy made my life . . .” on a 101-point scale from Much Worse to Much Better.

Respondents were then asked, “Have you ever contacted a pregnancy help center that did not refer for abortions?” with the option to answer yes, no, or unsure. Notably, this question was not strictly limited to the problematic pregnancy previously described, and the definition of “a pregnancy help center that did not refer for abortions” may have been unclear to some. Respondents choosing yes or unsure were then asked to rank the degree to which “The contact, counseling, or resources I received from the pregnancy help center made my life . . .” Much Worse to Much Better on another visual analog scale.

JASP 0.16.4 was used to analyze data distributions and to examine Pearson’s correlations between selected variables. Figures were created in Excel (Microsoft® Corp., Redmond, USA). The study design was approved by the Sterling Institutional Review Board (ID: 10225).

## Results

To obtain the required number of completed surveys, 1,161 persons in our selected gender and age range were invited by Cint to complete a survey on an unspecified topic. The first questions were demographic and allowed us to disqualify 122 respondents based on their reported age and gender. Of the remaining 1,039 eligible respondents, 39 (4%) failed to complete the survey, resulting in a 96% completion rate. Of the 1,000 women who completed the survey, 226 (22.6%) reported a history of abortion [16,17] and 275 (35.5%) of those who did not have a history of abortion reported experiencing a problematic pregnancy. Based on our abortion risk assessment (a score above 50 on at least one of the abortion risk scales), 112 (40.7%) of the women who reported a history of a problematic pregnancy had been at higher risk of abortion.

Demographic characteristics and the rate of contact with pregnancy help centers among the women who experienced at least one problematic pregnancy (excluding those who had abortions) are shown in Table 1 along with the percent for each category for all respondents and the related demographic data as reported for all adults in U.S. census data [16]. Comparisons to the census data reveal that the sample was somewhat more educated and more middle-income than the general population, which may be an artifact of both the subset of women aged 41-45 and possibly differences in the demographics of persons with a higher level of interest in completing surveys, such as those offered by Cint. Among the subset of respondents with problematic pregnancies who reported certainty that they had contact with a pregnancy care center, the rate of contact among women at a higher risk of abortion (17.9%) was twice that of problematic pregnancies at a lower risk of abortion (8.6%). However, considerable numbers of women from both groups (12.5% and 15.2%) were unsure if they had “contacted a pregnancy help center that did not refer for abortions.”

**Table 1:** Characteristics of the study population by percent *US Census data sources by region [18], by education [19], by household income [20], and by ethnicity [21]

Category	Characteristic	Lower abortion risk	Higher abortion risk	Combined	Entire survey sample	U.S. Census data for all adults*
		n (%)	n (%)	n (%)	n (%)	%
Race	Asian	6 (4%)	3 (3%)	9 (3%)	48 (5%)	6%
	Black	23 (14%)	19 (17%)	42 (15%)	150 (15%)	14%
	Hispanic	14 (9%)	14 (13%)	28 (10%)	137 (14%)	19%
	Other	13 (8%)	6 (5%)	19 (7%)	73 (7%)	2%
	White	107 (66%)	70 (63%)	177 (64%)	592 (59%)	59%
Educational attainment	Less than high school	10 (6%)	3 (3%)	13 (5%)	37 (4%)	11%
	High school graduate	57 (35%)	42 (38%)	99 (36%)	326 (33%)	26%
	University/higher education	76 (47%)	48 (43%)	124 (45%)	456 (46%)	49%
	Postgraduate education	20 (12%)	19 (17%)	39 (14%)	181 (18%)	14%
Income	$100,000 or more	41 (25%)	27 (24%)	67 (24%)	265 (27%)	36%
	$25,000 to $49,999	25 (15%)	17 (15%)	42 (15%)	148 (15%)	9%
	$50,000 to $74,999	43 (26%)	39 (35%)	82 (30%)	265 (27%)	19%
	$75,000 to $99,999	31 (19%)	18 (16%)	49 (18%)	188 (19%)	19%
	Less than $25,000	23 (14%)	11 (10%)	34 (12%)	134 (13%)	17%
Region	Midwest (IA, IL, IN, KS, MI, MN, MO, OH, ND, NE, SD, WI)	45 (28%)	20 (18%)	65 (24%)	209 (21%)	21%
	Northeast (CT, MA, ME, NH, NY, NJ, PA, RI, VT)	18 (11%)	12 (11%)	30 (11%)	144 (14%)	17%
	South (AL, AR, DC, DE, FL, GA, KY, LA, MD, MS, NC, OK, SC, TN, TX, VA, WV)	65 (40%)	63 (56%)	128 (47%)	437 (44%)	38%
	West (AK, AZ, CA, CO, HI, ID, MT, NM, NV, OR, UT, WA, WY)	35 (21%)	17 (15%)	52 (19%)	210 (21%)	24%
Pregnancy center contact	No	129 (79%)	75 (67%)	204 (74%)	-	-
	Unsure	20 (12%)	17 (15%)	37 (13%)	-	-
	Yes	14 (9%)	20 (18%)	34 (12%)	-	-

Table 2 shows the mean scores, standard deviations, and 95% confidence intervals (CIs) of the means. The highest mean was found on the scale measuring the risk of having an abortion if the respondent had not received support from others: 8.8 for the low abortion risk group and 61.9 for the high abortion risk group. Table 2 also shows that both groups retrospectively rated their pregnancy outcomes as having much improved their lives, with mean scores of 81.7 and 72.3 on a 100-point scale for those who were at lower risk of abortion and those at higher risk of abortion, respectively. A total of 71 (25.8%) respondents who had problematic pregnancies reported having certainly or possibly having had contact with a pregnancy help center, with 34 (12.4%) at lower risk and 37 (13.5%) at higher risk of abortion. On average, both groups gave similarly high ratings regarding that contact having made their lives better.

**Table 2:** Mean scale scores, mean 95% confidence intervals, and standard deviations segregated by lower- and higher-risk abortion groups

Scale (0-100)	Abortion risk	Valid	Mean	95% CI lower	95% CI upper	Std. deviation
Considered abortion	Lower	163	3.5	2.3	4.7	7.5
	Higher	112	57.2	51.3	63.1	32.0
	Combined	275	25.4	21.4	29.4	33.9
Researched abortion	Lower	163	3.3	2.2	4.4	7.3
	Higher	112	48.9	42.6	55.3	34.4
	Combined	275	21.9	18.1	25.7	31.9
At risk of abortion without the support of others	Lower	163	8.8	6.9	10.8	12.8
	Higher	112	61.9	56.0	67.8	32.0
	Combined	275	30.4	26.4	34.5	35.6
Pregnancy made life much better	Lower	163	84.8	81.7	87.9	20.1
	Higher	112	77.1	72.3	81.9	25.9
	Combined	275	81.7	79.0	84.4	22.9
Pregnancy center made life much better	Lower	34	71.1	65.3	77.0	17.5
	Higher	37	68.7	63.0	74.5	17.8
	Combined	71	69.9	65.8	74.0	17.6

The three abortion risk scales were strongly correlated with each other, as shown in Table 3. However, only two factors - having researched an abortion or being at higher risk of abortion without the support of others - were significantly correlated with having had contact with a pregnancy help center.

**Table 3:** Pearson’s correlations between abortion risk scales and pregnancy center contact * p < 0.05; ** p < 0.01; *** p < 0.001

Variable	Considered abortion	Researched abortion	At risk without support
Considered abortion	-	-	-
Researched abortion	0.805***	-	-
At risk without support	0.529***	0.553***	-
Pregnancy center contact	0.094	0.203***	0.199***

Table 4 shows the pregnancy outcomes relative to pregnancy center contact and abortion risk. The “% within column” percentages reveal that the odds of a healthy live birth were similar regardless of pregnancy center contact or abortion risk. For miscarriage and birth defects, the cell counts were too low to confidently identify any differences associated with pregnancy center contact, but there is at least an indication that women with pregnancies involving fetal defects may be more likely to have pregnancy center contact, perhaps to deal with the additional stress and planning associated with such pregnancies.

**Table 4:** Pregnancy outcomes relative to pregnancy center contact and abortion risk

Pregnancy outcome		Pregnancy center contact				Abortion risk		
		No	Unsure	Yes	Total	Low risk	High risk	Total
Healthy live birth	Count	165	29	28	222	133	89	222
	% within row	74.3%	13.1%	12.6%	100.0%	59.9%	40.1%	100.0%
	% within column	80.9%	78.4%	82.4%	80.7%	81.6%	79.5%	80.7%
	% of total	60.0%	10.5%	10.2%	80.7%	48.4%	32.4%	80.7%
Miscarriage, stillbirth, or other natural loss	Count	22	4	2	28	18	10	28
	% within row	78.6%	14.3%	7.1%	100.0%	64.3%	35.7%	100.0%
	% within column	10.8%	10.8%	5.9%	10.2%	11.0%	8.9%	10.2%
	% of total	8.0%	1.5%	0.7%	10.2%	6.5%	3.6%	10.2%
Birth with newborn defects	Count	12	3	4	19	9	10	19
	% within row	63.2%	15.8%	21.1%	100.0%	47.4%	52.6%	100.0%
	% within column	5.9%	8.1%	11.8%	6.9%	5.5%	8.9%	6.9%
	% of total	4.4%	1.1%	1.5%	6.9%	3.3%	3.6%	6.9%
Other	Count	5	1	0	6	3	3	6
	% within row	83.3%	16.7%	0.0%	100.0%	50.0%	50.0%	100.0%
	% within column	2.5%	2.7%	0.0%	2.2%	1.8%	2.7%	2.2%
	% of total	1.8%	0.4%	0.0%	2.2%	1.1%	1.1%	2.2%
Total	Count	204	37	34	275	163	112	275
	% within row	74.2%	13.5%	12.4%	100.0%	59.3%	40.7%	100.0%

**Figure 1:**
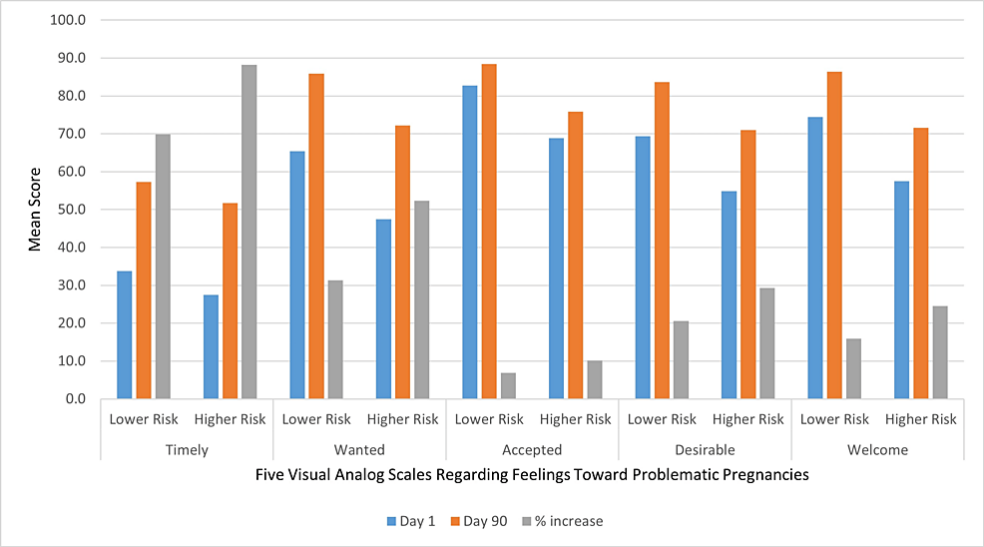
Changes in the mean score of feelings about problematic pregnancies from day 1 to day 90, comparing women at lower risk of abortion to those at higher risk of abortion

Figure 1 shows the mean score ratings of the problem pregnancy regarding timeliness, wantedness, acceptance, desirability, and welcomeness at the time women first realized they were pregnant and 90 days later, segregated into the lower and higher risk for abortion groups. The results show that across all measures, the lower risk of abortion group expressed more positive feelings about the problematic pregnancy compared to those in the higher risk of abortion group. However, positive feelings rose on each scale at faster rates for those at higher risk for abortion, suggesting that the difference in positive feelings was likely to diminish further as the pregnancy progressed. Both groups displayed the highest mean score for acceptance of the pregnancy, indicating a positive reaction. The most common negative reaction for both groups was that the pregnancy was untimely, but this was also the category that showed the most rapid improvement during the 90 days after first learning of the pregnancy. The second scale to improve most rapidly was wantedness. This may be due to the additional finding that, at least on average, these same problematic pregnancies were also rated higher, even at the time the pregnancy was first discovered, on the accepted, desirable, and welcomed scales. These dichotomies indicate that emotions related to problematic pregnancies can be complex and multilayered.

Table 5 shows distribution statistics for the three scales used to identify the degree of risk of having an abortion segregated by responses to the question of whether the respondent had contact with a “pregnancy help center that did not refer for abortions.” The results indicate that elevated risk for having an abortion without adequate support from others, more consideration of abortion as an option, and having researched an abortion were all more common among those women who reported having contacted a pregnancy help center compared to women who had not. Women who were unsure if they had had contact with such centers had abortion risk scores that tended to lie between the two other groups. Statistically significant differences are indicated whenever the 95% lower limit of the mean score of one group is higher than the 95% upper limit of another group. This test reveals that the mean scores for were unsure group were not significantly different from the other two groups. But the other two groups (yes versus no contact with a pregnancy help center) were significantly different on two of the abortion risk scales (at risk of abortion without the support of others, and for having researched abortion), but were not significantly different in regard to having considered abortion.

**Table 5:** Abortion risk scales segregated by contact with pregnancy help centers showing mean scores, 95% confidence interval of the means, and quartiles

Scale	Pregnancy center contact	Mean	95% CI lower	95% CI upper	25th percentile	50th percentile	75th percentile
Support abort risk	No	27.0	22.4	31.6	0.0	7.5	52.0
	Unsure	33.2	21.8	44.6	0.0	22.0	56.0
	Yes	48.2	36.7	59.7	20.0	44.0	76.3
Considered abortion	No	23.8	19.1	28.5	0.0	3.0	54.3
	Unsure	26.2	15.3	37.2	0.0	7.0	58.0
	Yes	33.9	22.8	45.0	1.3	26.0	58.8
Researched abortion	No	18.5	14.4	22.6	0.0	2.0	20.0
	Unsure	25.7	14.9	36.4	0.0	6.0	49.0
	Yes	37.9	25.9	50.0	3.3	24.0	63.5

For the women who answered yes or unsure in regard to having contact with a pregnancy help center, Table 6 shows the correlations between the scales measuring harm or benefit to respondents’ lives due to the pregnancy, contact with the pregnancy help center, and their risk of having an abortion as scored on the three abortion risk scales. The three abortion risk scales were strongly correlated to each other, but not to the benefits reported by contact with the pregnancy centers. However, higher scores relative to contact with the pregnancy center improving women’s lives were significantly correlated to a belief that the pregnancy made their lives much better, but it was not significantly correlated to any of the three abortion risk scales. This suggests that the positive effects were independent of abortion risk. More positive reports of the benefit of pregnancy to their lives were slightly, but significantly, negatively associated with having considered an abortion and having been at risk of abortion without the support of others. This suggests that women at higher risk of abortion reported slightly less improvement in their lives attributed to their pregnancy outcomes.

**Table 6:** Pearson’s correlations for the subset of respondents who reported contact with a pregnancy help center * p < 0.05; ** p < 0.01; *** p < 0.001

Variable	Pregnancy center made life much better	Pregnancy made life much better	Researched abortion	Considered abortion
Pregnancy center made life much better	-	-	-	-
Pregnancy made life much better	0.370**	-	-	-
Researched abortion	-0.079	-0.104	-	-
Considered abortion	-0.097	-0.162**	0.805***	-
At risk of abortion without the support of others	0.01	-0.175**	0.553***	0.529***

**Figure 2:**
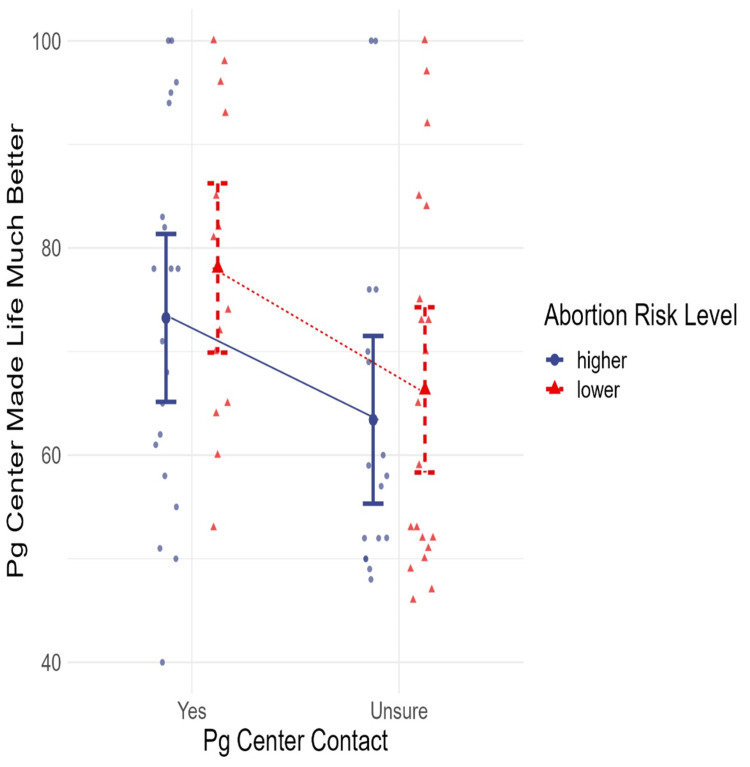
Scatter plot with means and standard error of means of the scale of the helpfulness of pregnancy center contact relative to the degree of abortion risk and certainty of having had contact with a pregnancy help center that does not refer for abortions pg: pregnancy

The distribution of scores ranking whether contact with a pregnancy center made the lives of women much worse or much better (on a scale from 0 to 100) is shown in Figure 2, with the results grouped by women who were certain that they had contact with a pregnancy help center which did not refer for abortions and those who were unsure, and by those in each group who were at higher or lower risk of having an abortion. This figure clearly shows that very few women reported that the contact worsened their lives (a score under 50), with the degree of any negative effects being very low, close to 50, with the single lowest rating being 40. The ratings of women who were at higher and lower risk of abortion were similar for both women who were sure and unsure of contact, but women at lower abortion risk were somewhat more likely to rate their experience more positively. Women who were more certain that they had contact with a pregnancy center were also more likely to report positive experiences than women who were unsure if they had contact with the type of center defined by the question.

## Discussion

Our exploratory study shows that women who face problematic pregnancies carried to their natural outcomes are likely to report an increase in positive feelings toward their pregnancies between the first day they learn of their pregnancies and the 90 days thereafter. This increase in positive feelings is significantly greater among the women at higher risk of having abortions, which is at least in part due to more negative feelings when they first learn of their pregnancies. At 41-45 years of age, both those who had been at lower and higher risk of abortion mostly reported that the pregnancy outcome had made their lives much better, with mean scores of 84.8 and 77.1, respectively. The single lowest evaluation was a score of 40, with all other scores at or above 50. Thus, while some critics have suggested that pregnancy help centers may harm women [15], our findings did not show any significant harm reported by the women themselves. Instead, most reported at least moderate to high levels of benefit to their lives, which they attributed to their pregnancy help center contact.

Our results also suggest that as many as one in four women facing problematic pregnancies (excluding those who reported having abortions) may have contact with a pregnancy help center. In our sample, 34 (12.4%) reported they had definitely contacted a pregnancy help center that did not make abortion referrals. Another 37 (13.5%) were uncertain if they had contacted an organization fitting that description. These percentages closely match the results of a prior study that identified women who were pregnant and considering abortions through Google Ads [11]. The relatively high level of uncertainty regarding whether or not a contacted organization did not refer for abortions was likely, at least in part, due to difficulties respondents faced in knowing for certain the abortion policies of every pregnancy center with which they had made contact. In addition, some women’s memories may have been unclear, especially if they had only one or two interactions with a community resource for pregnant women.

On average, both women with lower and higher risk of abortion who did have contact with pregnancy centers credited them with improving their lives. None reported that the contact had made their lives much worse.

A strength of our study is that it had a high participation rate in a sample that is reasonably representative of the national population of women 41-45 years of age, as confirmed by the findings that the number of women reporting abortions was very similar to the national estimates for women in this age group [17], and the proportion of women reporting contact with a pregnancy help center was very similar to that reported in the Google Ads study [11]. Another strength is that it explores a number of variables and metrics that have not previously been studied. These can help in the development of hypotheses that can be more carefully tested in future investigations.

The greatest weakness of this study is that it is entirely retrospective. Memories and feelings may change over time. Also, it is limited to women who are mostly nearing the end of their reproductive years, 41-45 years of age. This provides an approximation of lifetime exposure rates, but current rates of exposure may be lower or higher for women in younger age groups. To explore these effects across more age groups and time periods, it would be helpful if the variables examined in this study were included in longitudinal studies, such as the National Longitudinal Study of Adolescent to Adult Health.

Another limitation is that this survey was optimized to be completed in less than five minutes, which limited the range and complexity of survey items. Among these limitations, women who reported a history of abortion were presented with a different set of questions. As a result, this survey sheds no light on the experiences of women with a history of abortion who had contact with a pregnancy help center during, after, or prior to a pregnancy that ended in an abortion. Moreover, the very definition of a pregnancy help center that does not refer for abortions may have been unclear to some respondents, and in many cases, some may simply not have known what the abortion referral policies were of a pregnancy center with which they had made contact. Similarly, we included any woman who reported having experienced an “unplanned, mistimed, unwanted, or otherwise difficult pregnancy” in our category of women having faced a problematic pregnancy. But “otherwise difficult pregnancy” would also include planned pregnancies for which subsequent problems arose, whether medical, social, or economic. This broad inclusion criteria was intentional, for the purposes of this exploratory study, since these types of problems can both contribute to abortion risk and pregnancy center clientele. However additional research would be helpful to sort out the differences between pregnancies that were initially unplanned and those that were planned but later became problematic.

Another limitation is that survey length precluded asking every question relevant to each and every pregnancy experience. Women may have had more than one problematic pregnancy with different outcomes. In addition, we did not determine the abortion risk for each pregnancy. Indeed, we did not even specifically limit these questions to the problematic pregnancy the woman had in mind when she reported having an “unplanned, mistimed, unwanted, or otherwise difficult pregnancy.” The sequence and framing of the questions certainly implied that the abortion risk questions were in reference to the context of the problematic pregnancy reported but were not strictly limited to that one pregnancy. In future research, these questions should be asked in regard to each and every pregnancy that occurred in the period of observation.

Most ideally, a new prospective longitudinal study of a nationally representative sample of young women should be conducted to investigate the associations between all pregnancy outcomes and physical, psychological, familial, and socioeconomic health. The variables investigated should also include those factors that may lead to or away from problematic pregnancies and pregnancy losses (induced or natural), including access to and evaluations of the pregnancy help centers, abortion providers, and other resources of information and aid that may impact pregnancy outcomes and their associated effects.

## Conclusions

Over the course of their lifetimes, approximately one in four women will have experienced at least one induced abortion, and another one in four (with no history of induced abortions) will have faced problematic pregnancies (defined as unwanted, unplanned, untimely or otherwise difficult pregnancies) that they carried to their natural outcomes. Among those facing problematic pregnancies who report no history of induced abortions, approximately one-fourth reported that they sought or may have sought help from a pregnancy help center that does not refer for or provide abortions. The certainty of contact with a pregnancy center was approximately twice as high (17.9% versus 8.6%) among women who were at higher risk of abortion. Negative evaluations of contact with pregnancy help centers are uncommon. Instead, most women reporting contact with pregnancy help centers report that their contact with the pregnancy help center made their lives better to a moderate or high degree.

## References

[1] Finer LB, Zolna MR (2016). Declines in unintended pregnancy in the United States, 2008-2011. N Engl J Med.

[2] UNFPA UNFPA (2022). Nearly half of all pregnancies are unintended—a global crisis, says new UNFPA report. https://www.unfpa.org/press/nearly-half-all-pregnancies-are-unintended-global-crisis-says-new-unfpa-report.

[3] Helfferich C, Gerstner D, Knittel T, Pflügler C, Schmidt F (2021). Unintended conceptions leading to wanted pregnancies - an integral perspective on pregnancy acceptance from a mixed-methods study in Germany. Eur J Contracept Reprod Health Care.

[4] Aiken AR, Dillaway C, Mevs-Korff N (2015). A blessing I can't afford: factors underlying the paradox of happiness about unintended pregnancy. Soc Sci Med.

[5] Sable MR, Libbus MK (2000). Pregnancy intention and pregnancy happiness: are they different?. Matern Child Health J.

[6] Dalmijn EW, Visse MA, van Nistelrooij I (2024). Decision-making in case of an unintended pregnancy: an overview of what is known about this complex process. J Psychosom Obstet Gynaecol.

[7] Santelli J, Rochat R, Hatfield-Timajchy K (2003). The measurement and meaning of unintended pregnancy. Perspect Sex Reprod Health.

[8] Auerbach SL, Coleman-Minahan K, Alspaugh A, Aztlan EA, Stern L, Simmonds K (2023). Critiquing the unintended pregnancy framework. J Midwifery Womens Health.

[9] Aiken AR, Borrero S, Callegari LS, Dehlendorf C (2016). Rethinking the pregnancy planning paradigm: unintended conceptions or unrepresentative concepts?. Perspect Sex Reprod Health.

[10] Hussey LS (2013). Crisis pregnancy centers, poverty, and the expanding frontiers of American abortion politics. Politics and Policy.

[11] Cartwright AF, Tumlinson K, Upadhyay UD (2021). Pregnancy outcomes after exposure to crisis pregnancy centers among an abortion-seeking sample recruited online. PLoS One.

[12] Kimport K, Kriz R, Roberts SC (2018). The prevalence and impacts of crisis pregnancy center visits among a population of pregnant women. Contraception.

[13] Montoya MN, Judge-Golden C, Swartz JJ (2022). The problems with crisis pregnancy centers: reviewing the literature and identifying new directions for future research. Int J Womens Health.

[14] Bryant AG, Narasimhan S, Bryant-Comstock K, Levi EE (2014). Crisis pregnancy center websites: Information, misinformation and disinformation. Contraception.

[15] Borrero S, Frietsche S, Dehlendorf C (2019). Crisis pregnancy centers: faith centers operating in bad faith. J Gen Intern Med.

[16] Reardon DC, Longbons T (2023). Effects of pressure to abort on women’s emotional responses and mental health. Cureus.

[17] Reardon DC, Rafferty KA, Longbons T (2023). The effects of abortion decision rightness and decision type on women’s satisfaction and mental health. Cureus.

[18] (2024). US Census Bureau. https://www.census.gov/popclock/data_tables.php?component=growth.

[19] (2024). ACS 1-year estimates: S1501 educational attainment. https://data.census.gov/table/ACSST1Y2021.S1501?q=educational%20attainment&t=Education.

[20] (2024). HINC-01. Selected characteristics of households by total money income. https://www.census.gov/data/tables/time-series/demo/income-poverty/cps-hinc/hinc-01.html.

[21] (2024). QuickFacts: population estimates, July 1, 2023, (V2023). https://www.census.gov/quickfacts/fact/table/US/PST040223#PST040223.

